# Anti-TNFR1 targeting in humanized mice ameliorates disease in a model of multiple sclerosis

**DOI:** 10.1038/s41598-018-31957-7

**Published:** 2018-09-11

**Authors:** Sarah K. Williams, Richard Fairless, Olaf Maier, Patricia C. Liermann, Kira Pichi, Roman Fischer, Ulrich L. M. Eisel, Roland Kontermann, Andreas Herrmann, Babette Weksler, Nacho Romero, Pierre-Olivier Couraud, Klaus Pfizenmaier, Ricarda Diem

**Affiliations:** 1 Department of Neurology, University Clinic Heidelberg, Im Neuenheimer Feld 400, 69120 Heidelberg, Germany; 2 Institute of Cell Biology and Immunology, University of Stuttgart, Allmandring 31, 70569 Stuttgart, Germany; 3 Department of Molecular Neurobiology, Groningen Institute of Evolutionary Life Science, Faculty of Science and Engineering, University of Groningen, P.O. Box 11103, NL-9700 CC Groningen, The Netherlands; 4 Baliopharm AG, Eulerstr. 55, CH-4051 Basel, Switzerland; 5 Weill Medical College of Cornell University, New York, NY USA; 6 Department of Life, Health and Chemical Sciences, The Open University, Milton Keynes, MK7 6AA UK; 7 INSERM, U1016, Institut Cochin, Paris, France

## Abstract

Tumour necrosis factor (TNF) signalling is mediated via two receptors, TNF-receptor 1 (TNFR1) and TNF-receptor 2 (TNFR2), which work antithetically to balance CNS immune responses involved in autoimmune diseases such as multiple sclerosis. To determine the therapeutic potential of selectively inhibiting TNFR1 in mice with experimental autoimmune encephalomyelitis, we used chimeric human/mouse TNFR1 knock-in mice allowing the evaluation of antagonistic anti-human TNFR1 antibody efficacy. Treatment of mice after onset of disease with ATROSAB resulted in a robust amelioration of disease severity, correlating with reduced central nervous system immune cell infiltration. Long-term efficacy of treatment was achieved by treatment with the parental mouse anti-human TNFR1 antibody, H398, and extended by subsequent re-treatment of mice following relapse. Our data support the hypothesis that anti-TNFR1 therapy restricts immune cell infiltration across the blood-brain barrier through the down-regulation of TNF-induced adhesion molecules, rather than altering immune cell composition or activity. Collectively, we demonstrate the potential for anti-human TNFR1 therapies to effectively modulate immune responses in autoimmune disease.

## Introduction

Tumour necrosis factor (TNF), a master pro-inflammatory cytokine existing in both membrane-bound and soluble isoforms, plays a dominant role in the initiation and perpetuation of chronic inflammation^1,2^. It has been implicated in the pathology of many autoimmune diseases, where elevated TNF levels are reported. Similarly treatment of autoimmune diseases, such as rheumatoid arthritis, Crohn’s disease and psoriasis, with anti-TNF therapies have had successful outcomes^3^. One autoimmune disease where TNF plays a pivotal role is multiple sclerosis (MS) - a chronic inflammatory disease of the central nervous system (CNS), with a strong autoimmune inflammatory component accompanied by neurodegeneration^4^. Both serum and cerebrospinal fluid from MS patients contain elevated TNF^5^, which appear to correlate with symptom severity^6^. In addition, TNF and its two receptors, TNF-receptor 1 (TNFR1) and TNF-receptor 2 (TNFR2), are all up-regulated in MS lesions^7,8^.

The significance of the two TNF receptors has increasingly become clear since it is now appreciated that TNF mediates specific and often opposing effects through them. TNFR1, which is activated by both soluble and transmembrane TNF (with a higher affinity for soluble TNF)^9^, is implicated in promoting pro-inflammatory responses^10,11^, whereas, TNFR2, which is only fully activated by membrane-bound TNF, has been reported to mediate both neuroprotection and remyelination^12,13^. In a previous study, we demonstrated this differential effect using the experimental autoimmune encephalomyelitis (EAE) animal model of MS^14^. Here, we showed that whereas mice deficient in TNFR1 had a dramatically ameliorated disease course, TNFR2 deficient mice had more severe EAE. In turn, this information may explain the failure of a phase II anti-TNF therapeutic study carried out in relapsing-remitting MS patients^15,16^. Here, patients treated with non-selective TNF inhibitors had a worsening of neurological symptoms compared with those receiving placebo. Similarly, severe side effects have also been reported in approved anti-TNF treatment strategies, such as rheumatoid arthritis patients reporting the development of neurological symptoms, including demyelinating lesions^17,18^.

As a result, specific targeting of TNFR1 whilst leaving TNFR2 signalling unaffected might prove a more tolerable treatment regime for autoimmune diseases. This has been demonstrated in various EAE studies^19–22^. For example, in our previous study, we demonstrated that treatment with a mouse TNFR1-specific antagonistic antibody under both prophylactic and therapeutic treatment settings significantly ameliorated EAE^14^. However, the long-term aim of such studies is to develop treatment strategies with potential for human patients. With this in mind, here we investigate the potential of human TNFR1-selective antagonistic antibodies, ATROSAB and H398^23^, using humanized TNFR1 knock-in mice^24^.

## Results

### Treatment of EAE with anti-TNFR1 reduces disease severity

**Figure 1:**
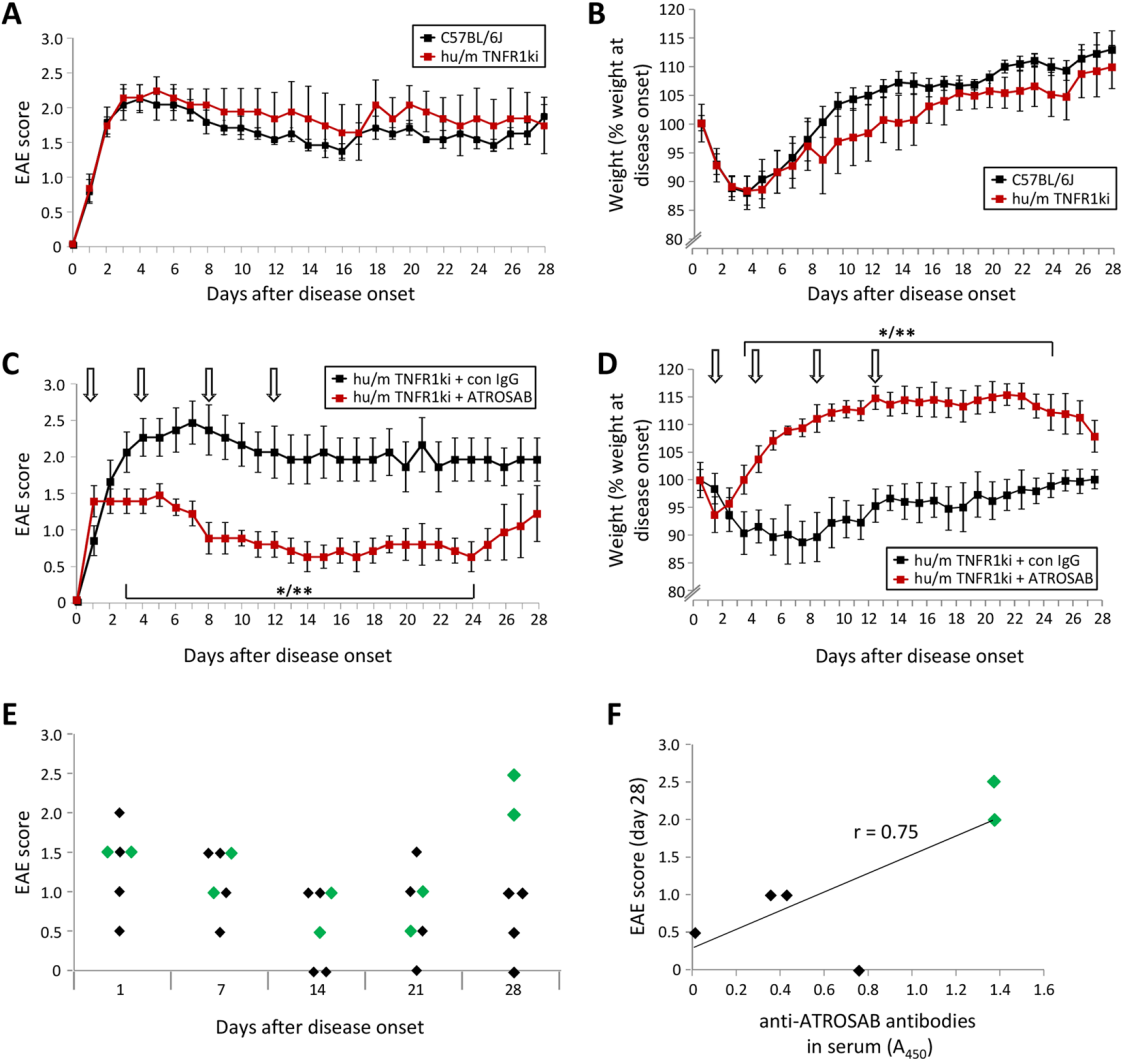
Treatment of EAE with ATROSAB reduces disease severity. (**A**) Wild type C57BL/6 J mice (n = 6) and hu/m TNFR1ki (n = 5) mice were both immunized with MOG_35−55_ and the course of EAE followed until 28 days after disease onset. No difference was seen between the courses of EAE in the two strains of mice. (**B**) Weight loss was also assessed, again revealing no differences between the mouse strains. (**C**) hu/m TNFR1ki mice were treated by intra-peritoneal injection with either 20 mg/kg ATROSAB (n = 6) or a corresponding control IgG (n = 5) on days 1, 4, 8 and 12 of manifest EAE and followed until day 28 of EAE. ATROSAB treatment led to a significant reduction in disease severity from the third day of EAE onwards. (**D**) Assessment of EAE-associated weight loss similarly revealed a significant improvement in the mice receiving 20 mg/kg ATROSAB. (**E**) Analysis of individual EAE scores of ATROSAB-treated mice from the experiment shown in panels C/D. Two mice (green diamonds) out of 6 appear to stop responding to treatment by the end of the experiment at day 28 of EAE. (**F**) Blood sera samples from these ATROSAB-treated mice were assessed for the presence of anti-ATROSAB antibodies by ELISA, demonstrating a positive correlation between anti-drug antibody levels and disease severity at experiment end (Pearson correlation coefficient of 0.75). All EAE studies were repeated, with one representative experiment being shown. ^*/**^p < 0.05/0.01.

In order to investigate the therapeutic potential of a human specific drug in a mouse model, chimeric human/murine TNFR-knock-in mice, in which the extracellular part of human TNFR1 is fused to the trans-membrane and intracellular region of mouse TNFR1, were used^24^. These mice were generated using a C57BL6 background, in which MOG immunisation typically results in a chronic progressive disease course^25,26^. Therefore, we initially sought to determine whether the presence of the chimeric TNFR1 would alter the progress of active EAE induced by immunization with MOG (amino acids 35–55). However, when immunized in comparison with wild type C57BL/6 J mice (WT), humanized TNFR1 knock-in mice (hu/m TNFR1-ki) displayed no difference in the day of onset (WT = 12.0 +/− 0.68; hu/m TNFR1-ki = 11.4 +/− 1.03), severity or disease course between the two lines (Fig. 1A). As a further indicator of animal well-being, the weight loss of mice was analysed, again showing no difference between the mouse lines (Fig. 1B).

EAE was then induced in hu/m TNFR1-ki mice via immunization with MOG_35−55_ and animals were injected intra-peritoneally with 20 mg/kg of either the human-specific TNFR1 antagonistic antibody ATROSAB, or an isotype control IgG (anti-hu epidermal growth factor receptor) on days 1, 4, 8 and 12 of manifest EAE. Time-points were chosen after disease manifestation in order to test the therapeutic potential of ATROSAB (average day of disease onset for all animals was day 13.7 +/− 0.5 post immunisation). ATROSAB-treated mice showed a highly significant reduction in disease severity in comparison to those treated with the control antibody, until 24 days after disease onset (Fig. 1C). In addition, ATROSAB significantly prevented EAE-associated weight loss, also until day 24 after the onset of disease (Fig. 1D).

Upon analysing the individual animal disease scores, it could be seen that in some animals (marked in green), the efficacy of ATROSAB treatment appeared to have ceased by the experiment end (Fig. 1E). In order to determine the reason for this increased disease severity in these ATROSAB-treated mice, we next performed an ELISA using immobilized ATROSAB to detect the potential presence of anti-drug antibodies (ADAs) which we hypothesized could interfere with treatment. A positive correlation could be seen between the level of anti-ATROSAB antibodies in the sera and the disease severity (r = 0.75, n = 6, p = 0.086; Fig. 1F), which occurred in approximately 30% of the mice analysed.

### Anti-TNFR1 treated mice have reduced demyelination and axonal damage

**Figure 2:**
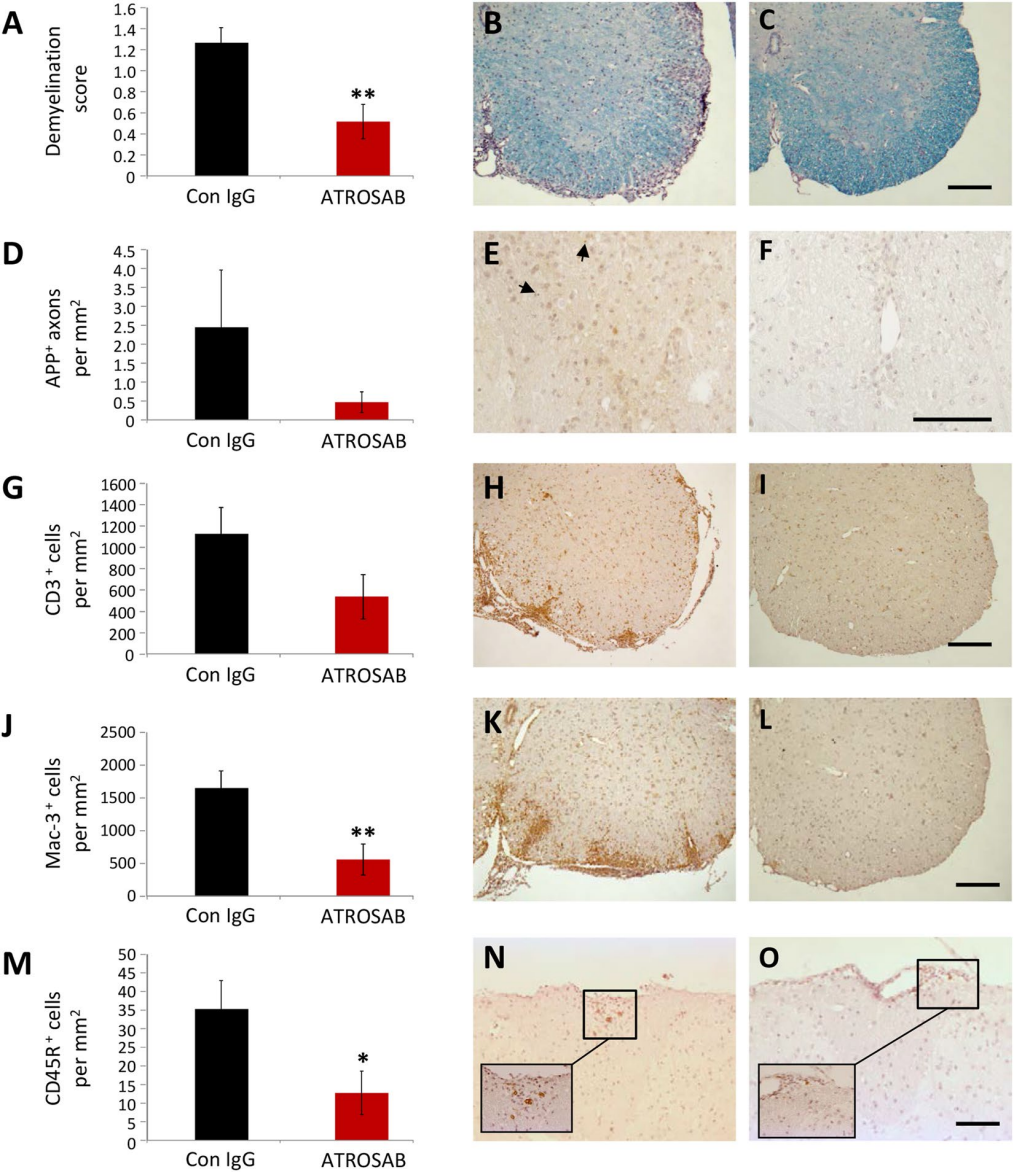
Treatment of EAE with ATROSAB improves histopathological alterations associated with the acute phase of EAE. Mice were treated with either 20 mg/kg ATROSAB (**C**,**F**,**I**,**L**,**O**) or a corresponding control IgG (**B**,**E**,**H**,**K**,**N**) on days 1 and 4 of EAE, and sacrificed during the acute phase of the disease on either day 5 or 6 of EAE. (**A**) Analysis of demyelination by staining of spinal cord sections with luxol fast blue (LFB) showed that ATROSAB significantly reduced spinal cord demyelination. (**D**) Acute axonal injury, as indicated by immunohistochemistry using an antibody again APP was reduced, though non-significantly, in the ATROSAB-treated group. (**G**) Infiltration of T cells into the spinal cord was reduced, again non-significantly, in the ATROSAB-treated group. As detected by immunohistochemistry, the presence of Mac-3^+^ activated microglia and macrophages in the spinal cord (**J**) was significantly reduced following ATROSAB treatment as was the infiltration of CD45R^+^ B cells (**M**). ^*^p < 0.05, ^**^p < 0.01. n = 6 per group. Scale bars C, I, L, O = 200 µm; F = 100 µm.

Two of the major pathological hallmarks of EAE are demyelination and axonal damage. In order to determine the histopathology of the spinal cord during acute EAE, mice were treated on days 1 and 4 of EAE with either ATROSAB or control IgG and perfused either 1 or 2 days later. Corresponding to the significantly reduced disease score at this time, ATROSAB treated mice, had significantly less spinal cord demyelination, as determined by analysis of LFB staining (Demyelination scores: control, 1.26 +/− 0.14; ATROSAB-treated, 0.52 +/− 0.13, t(10) = 3.85, p = 0.003; Fig. 2A–C). During this early phase of EAE, axonal damage is not very pronounced – however, analysis by immunohistochemistry to visualize accumulated amyloid precursor protein (APP), a marker of axonal injury since it is only detectable in axons with impaired axonal transport^27^, showed a reduction (though not significantly) in ATROSAB treated mice (APP^+^ axons per mm^2^: control, 2.45 +/− 1.51; ATROSAB-treated no ADAs, 0.39 +/− 0.21, U = 9.0, p = 0.18; Fig. 2D–F).

**Figure 3:**
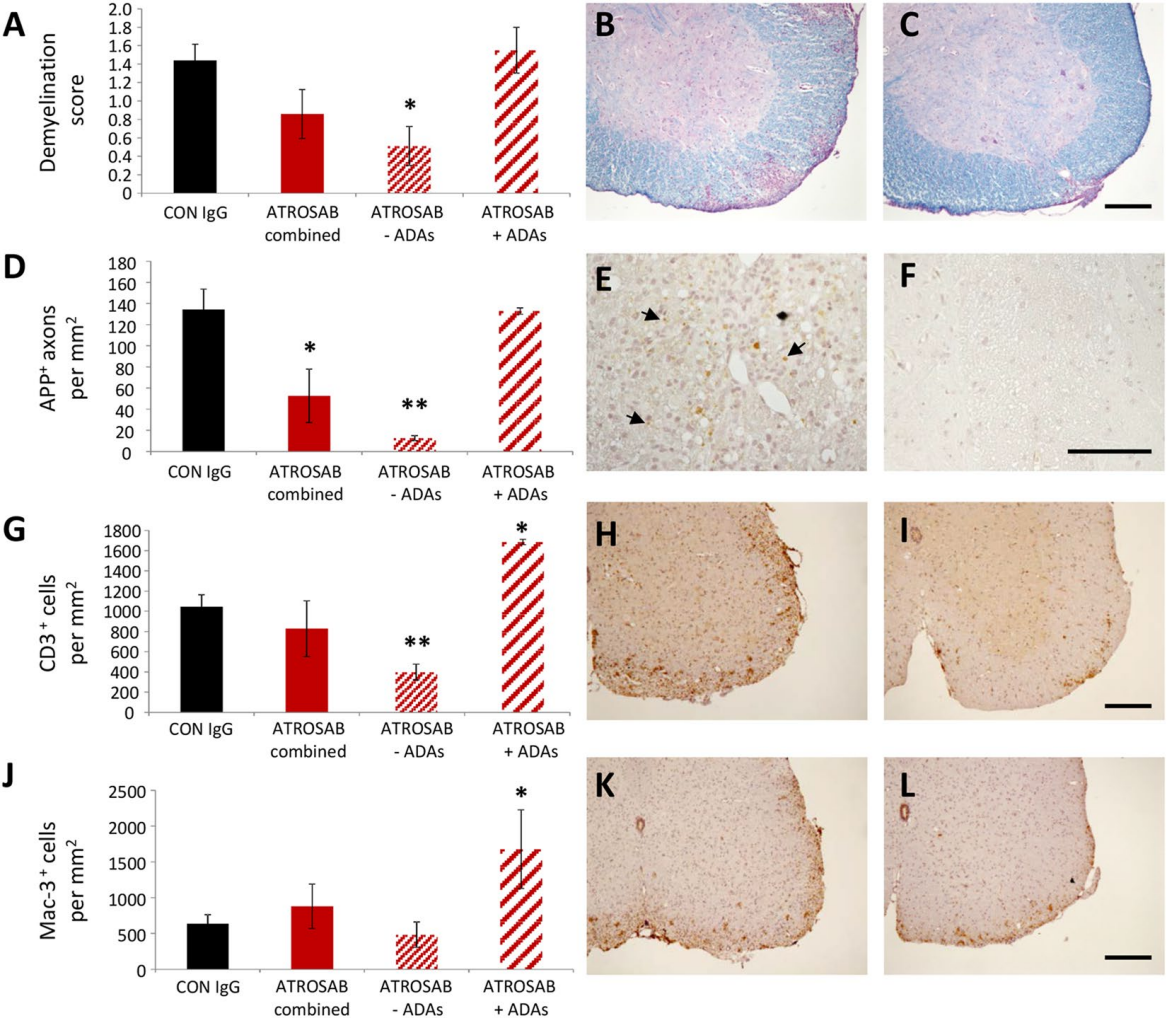
Treatment of EAE with ATROSAB improves histopathological alterations associated with the chronic phase of EAE. Mice were treated with either 20 mg/kg ATROSAB (**B**,**E**,**H**,**K**) or a corresponding control IgG (**C**,**F**,**I**,**L**) on days 1, 4, 8 and 12 of EAE, and sacrificed during the chronic phase of the disease on day 28 of EAE. (**A**) Analysis of demyelination by staining of spinal cord sections with LFB showed that ATROSAB significantly reduced spinal cord demyelination in mice not developing anti-drug antibodies (ADAs). (**D**) Axonal injury, as indicated by immunohistochemistry using an antibody against APP, was significantly reduced in the ATROSAB-treated group and was highly significant in treated mice which didn’t develop ADAs. Infiltration of CD3^+^ T cells (**G**) into the spinal cord was significantly reduced in ATROSAB treated mice which didn’t develop ADAs, but was significantly elevated in those that did. The presence of Mac-3^+^ activated microglia and macrophages (**J**) was not significantly altered between mice receiving either Con IgG or ATROSAB, although a significant elevation was seen in the sub-group which developed ADAs. ^*^p < 0.05, ^**^p < 0.01. n = 5 (con IgG) and 6 (ATROSAB). Scale bars C, I, L = 200 µm; F = 100 µm.

To determine the effect of ATROSAB on these parameters during chronic EAE, mice were treated with either ATROSAB or control IgG on days 1, 4, 8 and 12 of EAE and perfused on day 28 of EAE. Since at this later disease stage some mice developed ADAs, we analysed both the total group receiving ATROSAB, and further divided the group into those with or without ADAs. A significant reduction in demyelination was seen in ATROSAB-treated mice that didn’t develop ADAs (Demyelination score: control, 1.44 +/− 0.16; ATROSAB-treated, 0.51 +/− 0.17, t(9) = 1.75, p = 0.011; Fig. 3A–C). Furthermore at this later time point where axonal injury is much more abundant, there was a significant reduction in APP accumulation in the combined ATROSAB-treated group, which was more pronounced in the mice which didn’t develop ADAs (APP^+^ axons per mm^2^: control, 134.16 +/− 17.83; ATROSAB-treated no ADAs, 12.57 +/− 2.031, t(9) = 2.458, p < 0.001; Fig. 3D–F).

### Anti-TNFR1 treated mice have reduced inflammatory cell infiltration into the CNS

To investigate whether or not TNFR1 blockade also affected the presence of inflammatory infiltrates in the spinal cord, immunohistochemistry was performed during acute EAE (following two injections of ATROSAB). There was a trend towards reduced numbers of CD3^+^ T cells in the ATROSAB treated mice, compared to those in the control IgG group (control, 1126.42 +/− 203.62; ATROSAB-treated, 549.03 +/− 203.32, t(10) = 2.01, p = 0.07; Fig. 2G–I), and a significant reduction in the number of Mac-3^+^ activated microglia/macrophages (control, 1650.64 +/− 259.83; ATROSAB-treated, 561.65+/−212.66, U = 3.0, p = 0.008; Fig. 2J–L). There was also a significant reduction in the number of CD45R^+^ B cells within the spinal cord parenchyma during acute EAE, following ATROSAB treatment (control, 35.29 +/− 7.61; ATROSAB-treated, 12.73 +/− 5.36, U = 4.0, p = 0.026; Fig. 2M–O). At the later, chronic time point (EAE day 28), there was a highly significant reduction in the number of CD3^+^ T cells in mice that didn’t develop ADAs (control, 1044.96 +/− 107.59; ATROSAB-treated no ADAs, 398.96 +/− 64.23, t(9) = 0.67, p = 0.003; Fig. 3G–I) though no difference in the number of Mac-3^+^ activated microglia/macrophages (control, 636.42 +/− 115.69; ATROSAB-treated no ADAs, 482.19 +/− 145.93, t(9) = −0.68, p = 0.26; Fig. 3J–L) in the ATROSAB treated group. Interestingly, in mice that did develop ADAs, a significant elevation of both CD3^+^ and Mac-3^+^ cells were seen (ATROSAB-treated ADAs: CD3^+^, 1685.91 +/− 26.38, t(12) = 0.80, p = 0.023; Mac-3^+^, 1677.94 +/− 548.71, t(12) = 0.79, p = 0.034).

### Long term beneficial effects of TNFR1 inhibition

**Figure 4:**
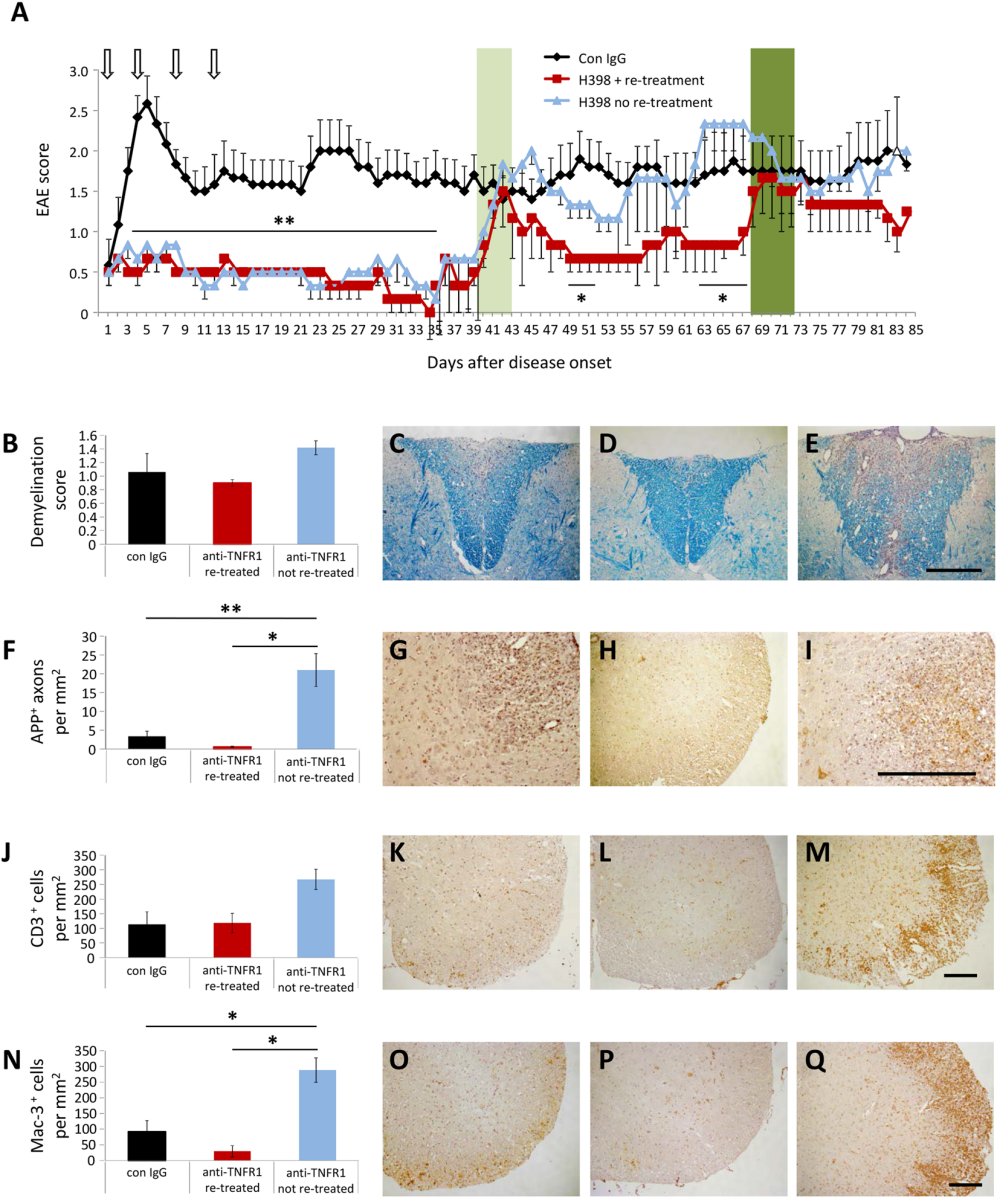
Long term treatment of EAE with anti-TNFR1 therapy is beneficial over an extended time frame. To prevent the occurrence of anti-drug antibodies, mice were treated with either H398, a mouse monoclonal antibody highly selective for human TNFR1 or control IgG on days 1, 4, 8 and 12 of manifest EAE. (**A**) H398-treated mice had reduced spinal cord deficits until approximately day 35 of EAE, at which point half the group received a further injection of 20 mg/kg H398 (re-treatment), and half received control IgG (no re-treatment) (relapse phase marked with light green bar). Those receiving H398 showed an improvement in disease severity which lasted approximately 20 days, after which mice underwent a further relapse (relapse phase marked with dark green bar) and were again treated with 20 mg/kg H398 or control IgG. Histopathological analyses of spinal cord were performed on all mice at day 85 of EAE and representative images shown from control IgG-treated mice (**C**,**G**,**K**,**O**), H398 + re-treatment (**D**,**H**,**L**,**P**) and H398 + no re-treatment (**E**,**I**,**M**,**Q**). Although demyelination (**B–E**) or T cell numbers (**J–M**) were not significantly affected by H398 re-treatment, mice which underwent a relapse following H398 treatment but no subsequent re-treatment had a significant elevation in the extent of axonal injury (**F–I**) and presence of activated microglia/macrophages (**N–Q**) than mice which underwent further H398 injections upon relapse (re-treated). ^*^p < 0.05, ^**^p < 0.01. n, control = 6; H398 + re-treatment = 3; H398 no re-treatment = 3. Scale bars = 200 µm.

To determine the long term beneficial effects of TNFR1 inhibition whilst avoiding the generation of ADAs, mice were treated with H398, the parental mouse monoclonal TNFR1 antagonistic antibody from which ATROSAB was derived by humanization^23,28^. The treatment regime followed was identical to that used for ATROSAB; mice were treated with either 20 mg/kg H398 or control IgG on days 1, 4, 8 and 12 of EAE. Blockade of TNFR1 with H398 again resulted in an amelioration of EAE, until approximately 35 days after the onset of EAE (Fig. 4A). After this time, all H398-treated mice experienced a discernible relapse with an increase in disease severity (marked in light green on Fig. 4A).

Following this increase in EAE severity, and on an animal-by-animal basis, half of the H398 treated group was re-treated with a single injection of 20 mg/kg H398 and the other half with 20 mg/kg of control IgG. Animals receiving this second H398 treatment showed another improvement in EAE which lasted for approximately 20 days, after which all mice underwent a second relapse (marked in dark green on Fig. 4A). Mice were once again treated with either H398 or control IgG (again consisting of a single injection of either 20 mg/kg H398 or 20 mg/kg of control IgG), at which time re-treatment with H398 appeared less effective at reducing the EAE score.

### Re-administration of a TNFR1 antagonistic antibody is beneficial in a long term study

Histopathological analyses of spinal cords were performed at day 85 after onset of EAE, comparing control treated mice (con IgG), with those receiving H398 treatment only during the first 12 days (no re-treatment) and those receiving a further re-treatment at approximately day 35 after onset of EAE and again at approximately day 60. No significant differences were seen between mice that received the initial H398 treatment followed by control IgG (i.e. not re-treated) and those receiving a re-treatment with H398 in demyelination (Demyelination score: re-treated, 0.90 +/− 0.03; not re-treated, 1.42 +/− 0.07, H(2) = 1.43, p = 0.53; Fig. 4B–E) or T cell infiltration (CD3^+^ cells, re-treated, 118.26 +/− 19.13; not re-treated, 266.82 +/− 24.49, H(2) = 4.31, p = 0.113; Fig. 4J–M). However, re-treatment appeared effective in preventing axonal damage (Fig. 4F–I). Mice receiving re-treatment had a reduced density of APP^+^ axons compared to mice receiving only control IgG (control, 3.41 +/− 1.32 APP^+^ axons per mm^2^; re-treated, 0.57 +/− 0.11; F(2,8) = 17.62, p = 0.69), however, H398-treated mice which were not re-treated had a significantly elevated APP^+^ axonal density compared to control IgG (not re-treated, 20.99 +/− 3.11, p = 0.001). This would be explained by the control IgG treated groups now reaching a chronic inactive lesion state whereby most axons have already been lost. In contrast, animals which were allowed to relapse due to no re-treatment were now undergoing active axonal damage as labelled by APP accumulation. In addition, there was a significant reduction in the number of Mac-3^+^ activated microglia/macrophages in the spinal cords of H398 re-treated mice (re-treated, 29.23 +/− 10.47; not re-treated, 288.72, F(2,8) = 8.75, p = 0.013; Fig. 4N–Q). In agreement with the APP data, animals allowed to relapse due to re-treatment only with control IgG had greater immune cell infiltration than animals only treated with control IgG from the start, suggesting, similar to the increase seen in mice developing ADAs (Fig. 3), that a rebound effect had occurred. Again, this argues that by day 85 EAE, the control treated mice had reached a state characterized by chronic inactive lesions: immune cell counts were now only about 100 cells/mm^2^ (Fig. 4J,N) compared with approximately 1000–2000 cells/mm^2^ during the acute stage (day 5/6 EAE; Fig. 2G,J) and 500–1500 during the chronic stage (day 28 EAE; Fig. 3G,J). Immune cell counts in relapsing, non-re-treated mice were still much lower than these ‘peak’ disease stages being around 250 cells/mm^2^.

### Inflammatory cells are unchanged following anti-TNFR1 treatment

**Figure 5:**
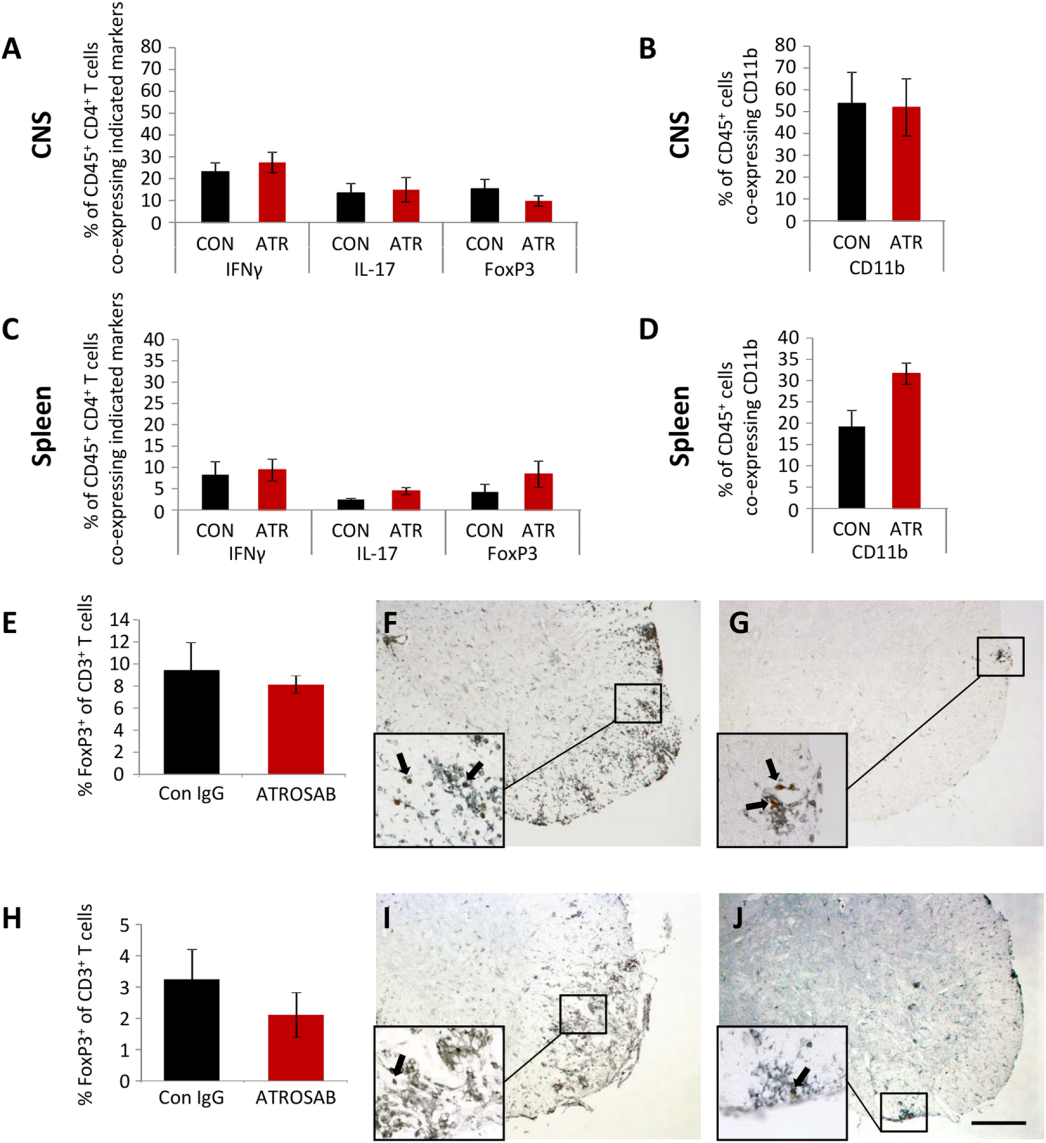
Peripheral and central immune cell phenotypes are not altered following anti-TNFR1 treatment. MNCs, isolated from the CNS (pooled brain and spinal cord; (**A**), and splenocytes (**C**) were collected on days 5 and 6 of acute EAE from mice treated with either ATROSAB or control IgG. Following gating for CD45^+^ CD4^+^ cells, FACS analysis was performed to determine the percentage of those co-expressing markers of either TH1 (IFNγ), TH17 (IL-17), or Tregs (FoxP3). In addition, following gating for CD45^+^ cells from the CNS (**B**) and spleen (**D**) on days 5 and 6 of acute EAE, cells were assessed for markers of monocytes (CD11b). No differences could be seen between cells from the two treatment groups (n = 4 mice per group). In addition, immunohistochemistry was performed to determine the percentage of CD3^+^ spinal cord infiltrates (black) co-expressing FoxP3 (brown), in order to identify Tregs. In comparison to control-treated animals, no difference was seen in the percentage of CD3^+^FoxP3^+^ cells in the ATROSAB-treated groups at either the acute (**F**–**G**) or chronic (**H**–**J**) disease stages. n = 6 per group. ATR, ATROSAB; CON, control IgG. Scale bar = 200 µm.

Due to the well-documented effects of TNFR signalling on cells of the immune system, we wished to see whether the beneficial effects of TNFR1 blockade in EAE were due to an alteration in the phenotype of leukocytes. To this end, T cells were isolated from both the CNS (pooled brain and spinal cord; Fig. 5A) and the peripheral immune system (spleen; Fig. 5C) of ATROSAB and control IgG-treated mice during acute EAE and, following gating for the CD45^+^ CD4^+^ pool, FACS analysis was used to determine the relative proportion of these cells co-expressing markers of TH1 (IFNγ), TH17 (IL-17), and Tregs (FoxP3). In addition, CD45^+^ leukocytes from both the CNS (Fig. 5B) and spleen (Fig. 5D) were analysed by FACS for the proportion of monocytes (CD11b). However, we did not see any effect of ATROSAB treatment on the proportion of CD45^+^ cells co-expressing these markers (Fig. 5, Suppl. Fig. 1).

Furthermore, we analysed the presence of Tregs within the spinal cord by immunohistochemistry. Again, ATROSAB treatment did not result in any change in the percentage of CD3^+^ cells co-expressing FoxP3 during either the acute (Fig. 5E–G) or chronic phases of EAE (Fig. 5H–J).

**Figure 6:**
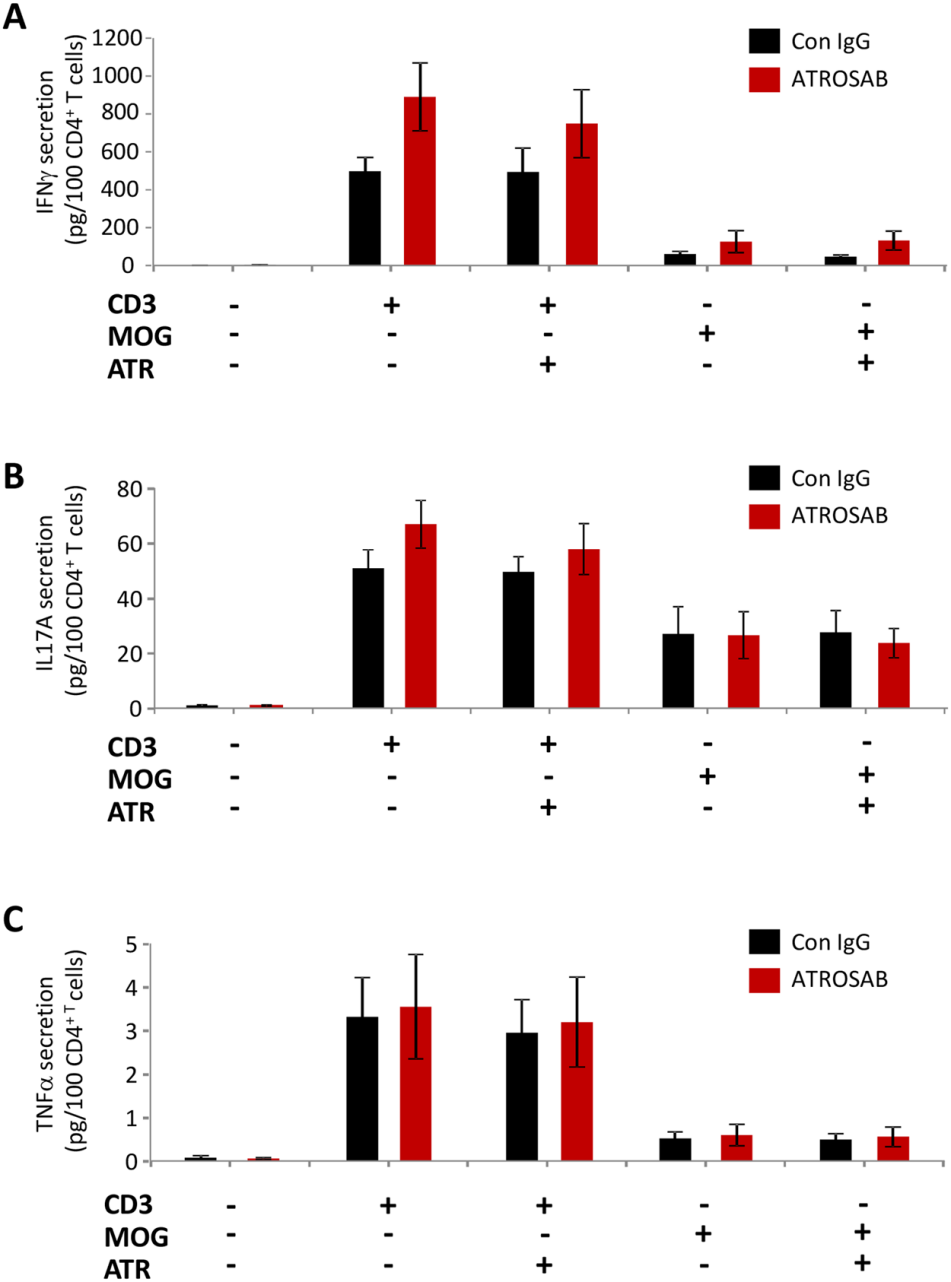
T cell cytokine secretion is unchanged by anti-TNFR1 treatment. T cells were isolated from lymph nodes following the second injection of ATROSAB/control IgG on days 5 or 6 of acute EAE. Following isolation and purification, T cells were re-stimulated with either anti-CD3 or MOG_35−55_ and ELISAs performed for IFNγ (**A**), IL17A (**B**) and TNFα (**C**). However, no differences were seen between T cells from both treatment groups in the secretion of either cytokine. n = 6 per treatment. ATR, ATROSAB.

In addition, we performed ELISAs on supernatant obtained from T cells isolated from acute EAE, to determine whether the amount of secreted IFNγ (Fig. 6A), IL-17A (Fig. 6B) or TNFα (Fig. 6C) were altered in mice treated with ATROSAB. However, following re-stimulation of isolated T cells with either CD3 or MOG_35−55_, no difference was seen in the levels of cytokines secreted by T cells isolated from either treatment group.

### Anti-TNFR1 treatment reduces endothelial cell expression of adhesion molecules and T cell adhesion

**Figure 7:**
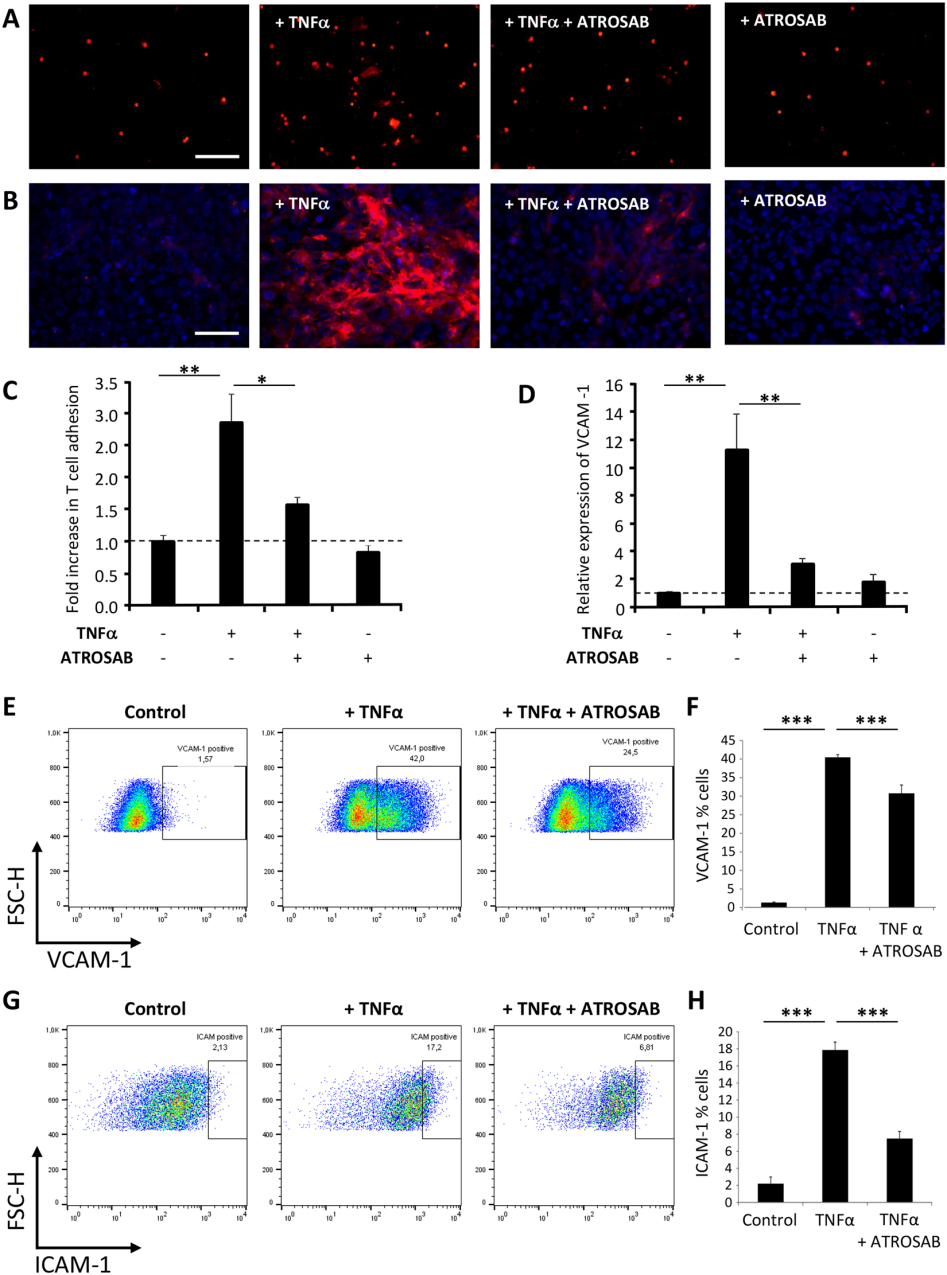
Anti-TNFR1 treatment reduces TNFα-induced T cell adhesion and endothelial cell adhesion molecule expression. (**A**) An adhesion assay of DiI-labelled T cells (red) was performed using a human brain endothelial cell line (hCMEC/D3) which was grown to confluency and pre-activated as indicated prior to the assay. (**C**) Quantification revealed that 24 hour pre-treatment with huTNFα significantly increased the number of adherent T cells, but not when co-treated with ATROSAB. (**B**) Similarly, 24 hour treatment of hCMEC/D3 cells with huTNFα resulted in a robust production of VCAM-1 (red), which was essentially blocked by ATROSAB co-treatment, as quantified in (**D**). Dapi counter-staining (blue) indicates the endothelial cell nuclei. Measurement of surface (**E**) VCAM-1 and (**G**) ICAM-1 expression on hCMEC/D3 cells was assessed by flow cytometry, with quantification given in (**F**,**H**), respectively, showing a significant reduction in TNFα-induced upregulation by co-incubation with ATROSAB. Scale bars = 100 µm. ^*^p < 0.05, ^**^p < 0.01, ^***^p < 0.001. (**C**,**D**), n = 4 per treatment, representative experiment of 2; (**F**), n = (control) 7, (TNFα) 7, and (TNFα + ATROSAB) 10; (**H**), n = (control) 3, (TNFα) 3, (TNFα + ATROSAB) 4.

Since immune cell infiltration was significantly reduced in animals receiving ATROSAB treatment, but immune cell composition and activity appeared unchanged, we next addressed whether anti-TNFR1 treatment could be affecting the ability of immune cells to enter the CNS. This was investigated by performing an adhesion assay using freshly isolated T cells labelled with DiI which were incubated on confluent monolayers of a human-derived endothelial cell line, hCMEC/D3. A human cell line was used in order to allow us to investigate the effect of the human TNFR1 specific reagent. Pre-treatment of endothelial cells with 100 ng/ml huTNFα for 24 hours prior to the adhesion assay resulted in a robust increase in the number of T cells able to adhere (2.84-fold increase +/− 0.45, H(3) = 13.06, p = 0.045; Fig. 7A,C). However, co-treatment of endothelial cells with TNFα and 100 µg/ml ATROSAB did not result in significantly increased T cell adhesion (only 1.56-fold increase compared to control cells +/− 0.11, p = 0.825).

Similarly, 24 hour treatment of hCMEC/D3 cells with huTNFα resulted in a robust increase in expression of VCAM-1 (11.26-fold increase +/− 2.60, H(3) = 11.73, p = 0.008; Fig. 7B,D), as assessed by immunocytochemistry. Again, co-treated cells (TNFα and ATROSAB) did not have significantly elevated VCAM-1 expression (only 3.02-fold increase compared to control, untreated cells +/− 0.43, p = 0.558). In order to quantify this further, surface expression of both VCAM-1 and ICAM-1 on hCMEC/D3 cells was measured by flow cytometry. Again, a robust increase in both VCAM-1 and ICAM-1 was seen following 24 hour incubation with huTNFα (VCAM-1, untreated, 1.31 +/− 0.16%; TNFα, 40.4 +/− 0.71%, Fig. 7F; ICAM-1, untreated 2.19 +/− 0.81%, TNFα, 17.86 +/− 0.93%, Fig. 7H). Upon co-incubation with ATROSAB both VCAM-1 and ICAM-1 expression were significantly reduced (VCAM-1, 30.73 +/− 2.29%; F(3,20) = 975.43, p < 0.001; ICAM-1, 7.50 +/− 0.82, F(2,7) = 78.23, p < 0.001), compared to TNFα alone. In order to assess if the expression levels of the T cell ligands for VCAM-1 and ICAM-1 were similarly regulated by TNFR1 modulation, we assessed the expression of CD49d (α chain of VLA-4, ligand for VCAM-1) and CD11a (αL integrin component of LFA-1, ligand for ICAM-1). CD4^+^ T cells were isolated from hu/m TNFR1ki mice at day 21 of EAE, treated with H398 or control IgG on days 1, 4, 8, 12 and 16 of EAE, and assessed by flow cytometry. No significant differences were seen in CD4^+^ T cell expression of either CD49d or CD11a between healthy mice, and mice with EAE receiving either treatment (Suppl. Fig. 2).

## Discussion

Here we have shown in a humanized TNFR1-ki mouse that inhibiting the effects of TNF signalling through TNFR1 using a human-specific TNFR1 antagonistic antibody has beneficial effects in EAE, a mouse model of MS. Furthermore, our data indicate that this effect is probably caused by reduced inflammatory cell infiltration into the CNS, possibly through a VCAM-1 mediated mechanism. This is in keeping with previous data by us and others that TNFR1 knock-out mice were much more resistant to EAE than wild type mice^14,29^. In the current study, although anti-TNFR1 treatment (4 injections up to day 12 following disease onset) appeared to be no longer effective after 30–40 days when a relapse was observed, we could additionally show that it was possible to re-treat mice, resulting in a significant extension of disease amelioration.

Anti-TNF therapy, although successful in inflammatory demyelinating animal models^30^, when trialled as a treatment strategy for MS during the Lenercept study, unexpectedly led to an exacerbation of symptoms in some patients^16^. The reason for this discrepancy between the animal models and humans remains unclear. However, it is interesting to note that anti-TNF antibodies showed varying efficacies between active and passive transfer models of EAE^31^, reflecting both the differing underlying mechanisms of these models and the pleiotropic nature of TNF. Furthermore, Lenercept, as a TNF receptor fusion protein, targets TNF itself without specificity for either receptor. Since then, our increased understanding of the complexity of TNF signalling has helped to explain these unforeseen results, as it is understood that when acting via TNFR1, TNF is generally pro-inflammatory, whereas TNF signalling via TNFR2 has been shown to be neuroprotective^1,2,12^. Therefore due to the essential role of TNFR2, we believe that a strategy of targeting TNFR1 specifically could avoid similar potentially unwanted outcomes.

Several different mechanisms of action for TNFR1 targeting have been proposed whereby TNF can mediate its effects through the modulation of diverse CNS cells. For example, constitutive expression of TNF in the CNS resulted in TNFR1-dependent apoptosis of oligodendrocytes^10^, whereas inhibition of TNFR1 signalling in mice with EAE through use of a selective blocker of soluble TNF, XPro1595, resulted in increased remyelination^22^. In addition, XPro1595 was reported to directly act on neurons during EAE since neuronal-specific deletion of the NF-ΚB signalling molecule, IKKβ, ablated its protective effects as assessed by EAE severity^20^.

However, in this study, we propose that anti-TNFR1 targeting is primarily acting to reduce disease severity by restricting immune cell infiltration into the CNS parenchyma. By acting on endothelial cells, the problem of the potential inability of TNF therapeutics to cross the BBB into the CNS (the so-called ‘lack of entry’ theory^32^) may not play such a crucial role in their ability to modulate disease. However, the relevance of this theory is also called into question by the opening of the BBB in demyelinating disorders, such as the EAE model, as well as reports of anti-TNF therapeutics leading to the occurrence of demyelinating symptoms following treatment for rheumatoid arthritis^33^.

Our proposal that anti-TNFR1 targeting acts to restrict immune cell infiltration is based on the following findings: 1) immune cell infiltrates were significantly reduced in ATROSAB-treated mice despite no changes in the immune cell compositions in the periphery (Fig. 5) nor the cytokine secretion profile of isolated T cells (Fig. 6); 2) ATROSAB reduced *in vitro* both expression of the endothelial cell adhesion molecules VCAM-1 and ICAM-1, as well as T cell adhesion to treated endothelial cells (Fig. 7), both necessary steps in the process of T cell extravasation; and 3) the rebound effect seen in mice following a relapse without re-treatment (Fig. 4) or possibly those developing potentially neutralising ADAs (Fig. 3) is similar to that reported in MS patients following withdrawal from Natalizumab treatment^34^. Natalizumab, one of the current therapies standardly offered to MS patients^35^, targets leukocyte extravasation through binding to the α4-subunit of α4β1 and α4β7 integrins inhibiting their adhesion to endothelial cell receptors, which also include VCAM-1^36^. Our observations also fit with other studies showing that endothelial cell expression of VCAM-1 and ICAM-1 is induced by TNFα signalling^37^ via TNFR1^38,39^ and that VCAM-1 expression is increased during EAE and subsequently reduced by anti-TNF targeting^40^.

Different antagonistic strategies for targeting TNFR1 appear to result in varying results regarding their mechanisms, despite all being effective in treating EAE. XPro1595, a dominant-negative soluble TNF subunit capable of combining with native TNF subunits to form an inactive heterotrimer, was reported to have negligible effects in reducing immune cell infiltration in one study whether administered therapeutically or prophylactically^20^. In another study using XPro1595, although a significant reduction in infiltrating macrophages was reported, no significant effects on either CD4 or CD8 T cell populations within the spinal cord were seen^22^. One major difference between our strategy and that of XPro1595, is that inhibition of soluble TNF, which preferentially binds TNFR1, would still leave trans-membrane TNF signalling to TNFR1 intact. In contrast, our approach was to directly block TNFR1 itself, as has been done in treatment studies of EAE mice treated with either R1antTNF, an antagonistic TNF mutant with selectivity for TNFR1^19^, or more recently with a nanobody-based selective inhibitor of TNFR1 (TROS)^41^, both resulting in a significant suppression of inflammatory cell infiltration. An additional benefit of our strategy resulted from the different pharmacokinetic properties of antibodies vs. TNF ligands, whereby 4 injections of H398 (given at 4 day intervals) were sufficient to reduce EAE disease for almost 40 days. Although, this long exceeds the half-life of both ATROSAB and H398 (2 +/− 0.3 days in hu/m TNFR-k/i mice^42^) subsequent re-treatment of relapsing mice was sufficient to achieve long-term protection from disease (Fig. 4).

Although we believe that anti-TNFR1 targeting might allow the complications reported in humans upon TNF targeting to be avoided, we demonstrate a potential risk that may follow discontinuation of treatment, due to the possibility of a rebound effect (as indicated both upon cessation of treatment (Fig. 4) and the development of potentially neutralising ADAs (Fig. 3)). More work will be required to allow a safe transition from bench to bed-side to be made. Improvements to the protocol might include a dual targeting of TNFR1 and TNFR2 as has been recently demonstrated in a model of NMDA-induced acute neurodegeneration, in order to obtain the benefits of both inhibiting deleterious TNFR1 activation, whilst promoting potentially protective TNFR2 signalling^24^. This might achieve both a restriction of lymphocytic extravasation across the BBB, as well as immunomodulation in the periphery.

In conclusion, anti-TNFR1 targeting with antibodies is an effective strategy for reducing the neurological deficits arising during EAE in mice. Through the use of humanized transgenic mice, we demonstrate that this approach has the potential for translation into a therapy for MS patients in the future.

## Materials and Methods

### Animals

Female C57BL/6 J mice (RRID: IMSR_JAX.000664) were obtained from specific pathogen free rooms of Charles River (Sulzfeld, Germany). Humanized TNFR1 knock-in (hu/m TNFR1-ki) mice were generated by Ozgene Pty Ltd as previously described^24^, and were kept under environmentally controlled conditions in the absence of pathogens. All animal work was performed in accordance with European and German animal protection law with approval from the ‘Regierungspräsidium’ in Karlsruhe, Germany.

### Induction and evaluation of EAE and treatment of mice

EAE was induced as previously described^14,43^. Briefly, mice were immunized sub-cutaneously with 300 µg MOG_35−55_ and received intra-peritoneal injection of 300 ng pertussis toxin immediately afterwards and again 2 days later. Mice were weighed and scored on a daily basis, with disease severity assessed using a scale ranging from 0 to 5: 0, no clinical disease; 0.5, distal paresis of the tail; 1.0, complete paralysis of the tail; 1.5, paresis of tail and mildly impaired righting reflex; 2.0, gait ataxia and severely reduced righting reflex; 2.5, bilateral severe hind limb paresis; 3.0, complete bilateral hind limb paralysis; 3.5, complete bilateral hind limb paralysis and forelimb paresis; 4, hind and fore limb paralysis; 5, moribund state or death. Mice received intra-peritoneal injections with either the human TNFR1-selective antagonistic antibodies ATROSAB^23,44^ or H398^45^ on the days of EAE as stated. Control mice received appropriate species-specific control IgG (anti-hu epidermal growth factor receptor antibody Cetuximab^46^ (RRID: AB_24896053) for ATROSAB, or mouse IgG (Sigma Aldrich, St. Louis, MO, USA; RRID: AB_253291), for H398.

### Spinal cord histopathology

Mice received an overdose of ketamine/xylazine and were transcardially perfused with 4% PFA in PBS. Spinal cords were dissected, processed for paraffin-embedding and 0.5 µm transverse sections were cut, with 10 sections per stain taken at regular intervals to cover the whole spinal cord. LFB staining was performed in order to assess demyelination, as previously described^14^. Briefly, the degree of demyelination was evaluated semiquantitatively using the following scoring system: 0.5, traces of perivascular or subpial demyelination; 1, marked perivascular or subpial demyelination; 2, confluent perivascular or subpial demyelination; 3, demyelination of half spinal cord cross section; and 4, transverse myelitis. For immunohistochemistry, antigen retrieval was performed by incubating tissue sections in heated (~80 °C) 0.2% citrate buffer (pH 6.0) for 15 mins, before being left to cool. Antibodies against Mac-3 (1:200, BD Biosciences, San Jose CA, USA; RRID: AB_393587), CD3 (1:150, Dako, Glostrup, Denmark; RRID: AB_2335677), CD45R/B220 (1:50, BD Biosciences; RRID: AB_393581), FoxP3 (1:100, eBioscience, San Diego, CA, USA; RRID: AB_467575) and APP (1:2500, Millipore, Darmstadt, Germany; RRID: AB_94882) were used to detect activated microglia/macrophages, T cells, B cells, regulatory T cells, and damaged axons, respectively. For all histopathological and immunohistochemical investigations, a minimum of 10 sections taken throughout the length of the spinal cord were quantified. All microscopy was performed on an Eclipse 80i upright microscope with 2×, 10×, 20×, 40× or 60× objectives and fitted with a DXM1200C camera (Nikon, Shinagawa, Tokyo, Japan).

### Isolation of CNS mononuclear cells

CNS mononuclear cells (MNCs) were isolated using a modified method based on previously published protocols^47,48^. Brains and spinal cord were removed from mice at acute EAE (day 5/6 after onset of disease) and digested with 0.05% collagenase D (Roche Diagnostics GmbH, Mannheim, Germany), 10 µg/ml DNase I (Sigma-Aldrich), TLCK trypsin inhibitor (Sigma-Aldrich) and 10 mM HEPES pH 7.4 (Sigma-Aldrich) in HBSS (Sigma-Aldrich) for 1 hr at 37 °C. Cells were mechanically dissociated with a 100 µM cell strainer (Corning, Kaiserslautern, Germany) and resuspended in 1.088 mg/ml Percoll (Easycoll; Biochrom AG, Berlin, Germany). MNCs were isolated using a Percoll gradient of differing densities: 0 g/ml, 1.072 g/ml, 1.088 g/ml and 1.124 g/ml, and centrifuged at 1250 g at 20 °C for 45 mins without brakes. MNCs were then harvested from the interface between the Percoll densities of 1.072 g/ml and 1.088 g/ml.

### Isolation of spleenocytes and spleen T cells

Spleens were isolated and mechanically dissociated using a 70 μm cell strainer, washed in ice cold PBS and then erythrolysis was performed using ACK lysing buffer (Thermo Fisher Scientific, Waltham, MA, USA). For some experiments, T cells were then further isolated using a Pan T cell Isolation Kit according to the manufacturer’s instructions (Miltenyi Biotech GmbH, Bergisch Gladbach, Germany).

### Human brain endothelial cell line

hCMEC/D3 cells^49,50^ (RRID: CVCL_U985) were cultured on collagen type I-coated flasks in EGM-2 MV medium (Lonza, Walkersville, MD, USA) supplemented with (v/v) 0.025% rhEGF, 0.025% VEGF, 0.025% IGF, 0.1% rhFGF, 0.1% gentamicin, 0.1% ascorbic acid, 0.04% hydrocortisone, and 2.5% FBS. Cells were maintained at 37 °C in 95% air and 5% CO_2_ and used for experiments between passages 29 and 33. For experiments, confluent hCMEC/D3 cells were reseeded on collagen I-coated coverslips, regrown to confluence, before switching to the experimental media (EGM-2 MV supplemented with all the above growth factors except for IGF and VEGF) for 30 minutes. Cells were then treated for 24 hours in experimental media supplemented with human rTNFα (100 ng/ml, R&D Systems, Minneapolis, MN, USA) and/or ATROSAB (100 µg/ml) as indicated, before either fixation with 4% PFA, being used for adhesion assays, or harvested for flow cytometry. For immunocytochemistry, fixed cultures were incubated for 30 minutes with 10% normal goat serum in PBS, followed by primary (VCAM-1; 1:100, Abcam; RRID:AB_2721053) and a Cy-3 conjugated secondary antibody (1:400, Jackson ImmunoResearch Labs, Ely, UK; RRID:AB_2338006). After washing, preparations were mounted in DAPI-containing Vectashield medium (Vector laboratories, Burlinghame, CA, USA). For quantification of VCAM-1 expression, images were analysed with ImageJ analysis software (NIH, USA), experiments repeated at least four times using five random areas per coverslip and measured for fluorescence intensity using similarly exposed images.

### Cytokine release by primary lymphocytes

Inguinal lymph nodes of hu/m TNFR1-k/i mice (EAE day 5/6) were isolated and mashed through a 50 μm cell strainer with 10 ml PBS containing 1% BSA and 0.5 mM EDTA. Cells were centrifuged (300 × g, 5 min) and washed once with the same buffer. For general stimulation, 1 × 10^5^ viable cells (determined by trypan blue staining) were plated on anti-CD3 (clone 17A2 BioLegend, San Diego CA, USA)-coated (6 h at 4 °C, 2 μg/ml) 96-well (U) plates and cultivated for 3 days in RPMI 1640 supplemented with 10% mouse serum (Sigma-Aldrich), 50 μM β-mercaptoethanol (Life Technologies, Karlsbad, CA, USA) and penicillin/streptomycin (Sigma-Aldrich). For specific re-stimulation, cells were plated onto uncoated 96-well plates and cultivated similarly in presence of 50 µM MOG_35−55_. To analyse whether TNFR1-inhibition affects cytokine release, ATROSAB (100 µg/ml) was added to some wells. To determine the cytokine release of the activated lymphocytes, the culture medium was collected and analysed by ELISAs specific for IL-17A, IFNγ and TNFα, respectively, according to the instructions of the manufacturer (BioLegend). After incubating with TMB substrate and terminating the reaction with 1 M H_2_SO_4_, the absorbance at 450 nm was determined with an absorbance reader (Multiskan FC; Thermo Fisher Scientific). The amount of released cytokines was determined with the provided standard and calculated using Microsoft Excel and GraphPad Prism 4 (Graph-Pad, La Jolla, CA, USA). In order to quantify the concentration of released cytokines with respect to the number of CD4^+^ T cells in the mixed lymphocyte culture at the start of cultivation, a sample of the lymphocytes was taken for analysis of the CD4^+^ population by flow cytometry (MACS-Quant, Miltenyi) following labelling with anti-CD4-FITC (Miltenyi).

### Flow cytometry

CNS MNCs and spleenocytes were stimulated with 1 µg/ml ionomycin (Sigma-Aldrich), 5 µg/ml Brefeldin A (Sigma-Aldrich) and 20 ng/ml PMA (Sigma-Aldrich) in RPMI (RPMI-1640; PAN Biotech, Aidenbach, Germany) supplemented with 2 mM L glutamine (Thermo Fisher Scientific), 10% FBS (Thermo Fisher Scientific), 100 U/ml penicillin, 0.1 mg/ml streptomycin (Sigma-Aldrich), 25 mM HEPES pH 7.4, 1 mM sodium pyruvate (PAA, Cambridge, UK), 0.1 mM non-essential amino acids solution (Lonza, Slough Wokingham, UK), 5 × 10^−5^ M 2-mercaptoethanol (Sigma-Aldrich) at a density of 0.1 × 10^6^ cells/ml for 4 hrs prior to staining. Cells were stained with the following antibodies against surface antigens: Pacific Blue anti-mouse CD4 (RM4-5, Biolegend; RRID: AB_2721777), Pacific Orange anti-mouse CD45 (MCD4530, Molecular Probes, Life Technologies; RRID: AB_2539700), and FITC anti-mouse CD11b (M1/70 eBioscience; RRID: AB_464934). For intracellular staining, cells were fixed and permeabilized using Cytofix/Cytoperm® (BD Biosciences) according to the manufacturer’s protocol, followed by incubation with fluorescently labeled antibodies against intracellular molecules for 30 min at 4 °C: APC anti-mouse FoxP3 (FJK-16s, eBioscience; RRID: AB_469457), APC-Cy7 anti-mouse IL17a (TC11-18H10, BD Biosciences; RRID: AB_2034016) and phycoerythrin anti-mouse IFNγ (XMG1.2, eBioscience; RRID: AB:465412). For analysis of T cell integrin expression, antibodies against CD49d (PE conjugated, clone 9C10, BD Biosciences; RRID: AB_396693) and CD11a (APC conjugated, M17/4, eBioscience; RRID: AB:11217481) were used.

hCMEC/D3 cells were harvested in PBS containing 0.02 mM EDTA and 0.4 mg/ml collagenase (Sigma-Aldrich). Cells were then stained with antibodies against either human VCAM-1 (CD106-PE, STA, eBioscience; AB_10854126) or ICAM-1 (CD54-APC, HA58, eBioscience; AB_10718240). Cells were then fixed in 1% PFA, and kept in the dark until analysis. Flow cytometry acquisition was performed on a FACSCanto II (BD Biosciences) using the BD FACSDiva software. All data was analysed using FlowJo software.

### Adhesion assay

Following treatment of hCMEC/D3 cells for 24 hours as indicated, freshly prepared T cells from the spleens of naïve mice were labelled with 5 µM DiI (Sigma-Aldrich; 1 ml per 10^6^ cells) for 10 minutes. 5 × 10^5^ T cells were added to each coverslip of hCMEC/D3 cells and then left 3 hours at 37°C 5% CO_2_ to adhere, before washing off unbound cells. Coverslips were then fixed in 4% PFA, and DiI-labelled cells were quantified under a microscope.

### Determination of anti-drug antibodies in serum

ELISA plates (Greiner, Frickenhausen, Germany) were coated with ATROSAB at 1 μg/ml in PBS and incubated at 4 °C overnight. Residual binding sites were blocked with 2% skimmed milk powder in PBS at room temperature (RT) for 2 h. The serum of ATROSAB-treated mice was diluted in 2% skimmed milk powder in PBS and added to the plates for 2 h at RT. Bound proteins were detected with HRP-conjugated anti-mouse IgG antibodies (diluted 1:10,000; incubation for 1 h at RT), followed by incubation with TMB substrate solution. The reaction was stopped by addition of 1 M H_2_SO_4_, and the absorbance at 450 nm was determined. Data were analysed using the software GraphPad Prism 4. Between each step, non-bound proteins were removed by washing four times with 0.005% Tween-20 in PBS.

### Experimental design and statistical analyses

Experiments were performed only on female mice of the same age (6–8 weeks old) which were housed under the same conditions. Hu/m TNFR1-ki were initially screened against C57BL/6 J mice for EAE to control for potential differences in their disease progression (Fig. 1A,B). ATROSAB EAE experiments were performed on 5 separate occasions, with a total of 51 hu/m TNFR1-ki treated with control antibody and 52 with ATROSAB. The H398 EAE experiment was performed once with 6 hu/m TNFR1-ki receiving control antibody, and 6 hu/m TNFR1-ki receiving H398 as indicated. All data are presented as mean +/− SEM. Statistical comparisons were made using SigmaPlot 12 (Systat Software GmbH, San Jose, CA, USA). Data was assessed for normality using the Shapiro-Wilk Test, followed by either a Mann-Whitney or a two-tailed student’s t-test for comparing two experimental groups, or the one way analysis of variance (ANOVA) with post-hoc Dunnett’s or Tukey’s test, or Kruskal-Wallis test with post-hoc Dunn’s analysis for multiple group comparisons. Correlations were calculated using the Pearson-Moment Correlation Coefficient using Sigma Plot. A p value of <0.05 was considered to be statistically significant.

## Electronic supplementary material

Supplementary Figures

## Data Availability

The datasets generated during and/or analysed during the current study are available from the corresponding author on reasonable request.
