# Supplementary material for: Anti-TNFR1 targeting in humanized mice ameliorates disease in a model of multiple sclerosis
Source: Sci Rep. 2018 Sep 11;8:13628. doi: 10.1038/s41598-018-31957-7 (PMC6133964; doi:10.1038/s41598-018-31957-7)
Supplement: Supplementary file 1 — Supplementary Figures [file 41598_2018_31957_MOESM1_ESM.pdf]

Supplementary Information.

Anti-TNFR1 targeting in humanized mice ameliorates disease in a model of multiple sclerosis

Sarah K. Williams[1*], Richard Fairless[1], Olaf Maier[2], Patricia C. Liermann[1], Kira Pichi[1], Roman Fischer[2], Ulrich L. M. Eisel[3], Roland Kontermann[2], Andreas Herrmann[4], Babette Weksler[5], Nacho Romero[6], Pierre-Olivier Couraud[7], Klaus Pfizenmaier[2] and Ricarda Diem[1]

[1]Dept. of Neurology, University Clinic Heidelberg, Im Neuenheimer Feld 400, 69120 Heidelberg, Germany, [2]Institute of Cell Biology and Immunology, University of Stuttgart, Allmandring 31, 70569 Stuttgart, Germany, [3]Department of Molecular Neurobiology, Groningen Institute of Evolutionary Life Science, Faculty of Science and Engineering, University of Groningen, P.O. Box 11103, NL-9700 CC Groningen, The Netherlands, [4]Baliopharm AG, Eulerstr. 55, CH-4051 Basel, Switzerland, [5]Weill Medical College of Cornell University, New York, NY, USA, [6]Department of Life, Health and Chemical Sciences, The Open University, Milton Keynes MK7 6AA, UK, [7]INSERM, U1016, Institut Cochin, Paris, France

Running title: ATROSAB treatment of EAE

***Corresponding author**
Sarah K. Williams, PhD
Department of Neurology
University of Heidelberg
Otto-Mayerhof-Zentrum (OMZ)
Im Neuenheimer Feld 350
69120 Heidelberg, Germany
phone: +49-6221-56-35688
fax: +49-6221-56-5837
email: s.williams@dkfz-heidelberg.de

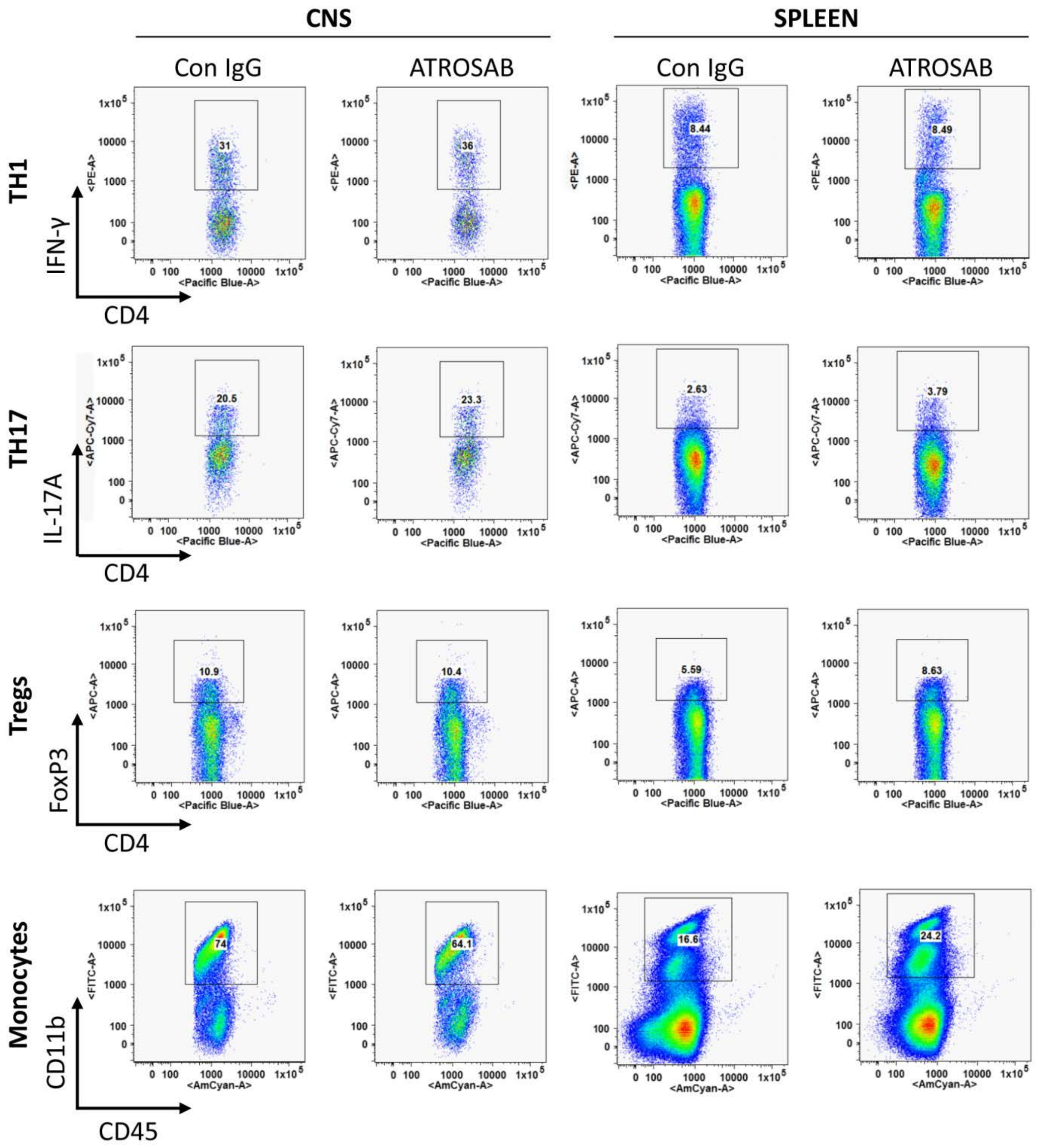

Supplementary Figure 1. Representative dot plots for FACS data given in Figure 5 (panels A-D). Cells labelled against intracellular IFN-γ (TH1), IL-17A (TH17) and FoxP3 (Tregs) were gated for CD45 and CD4 positivity. Cells labelled for CD11b (monocytes) were gated for CD45.

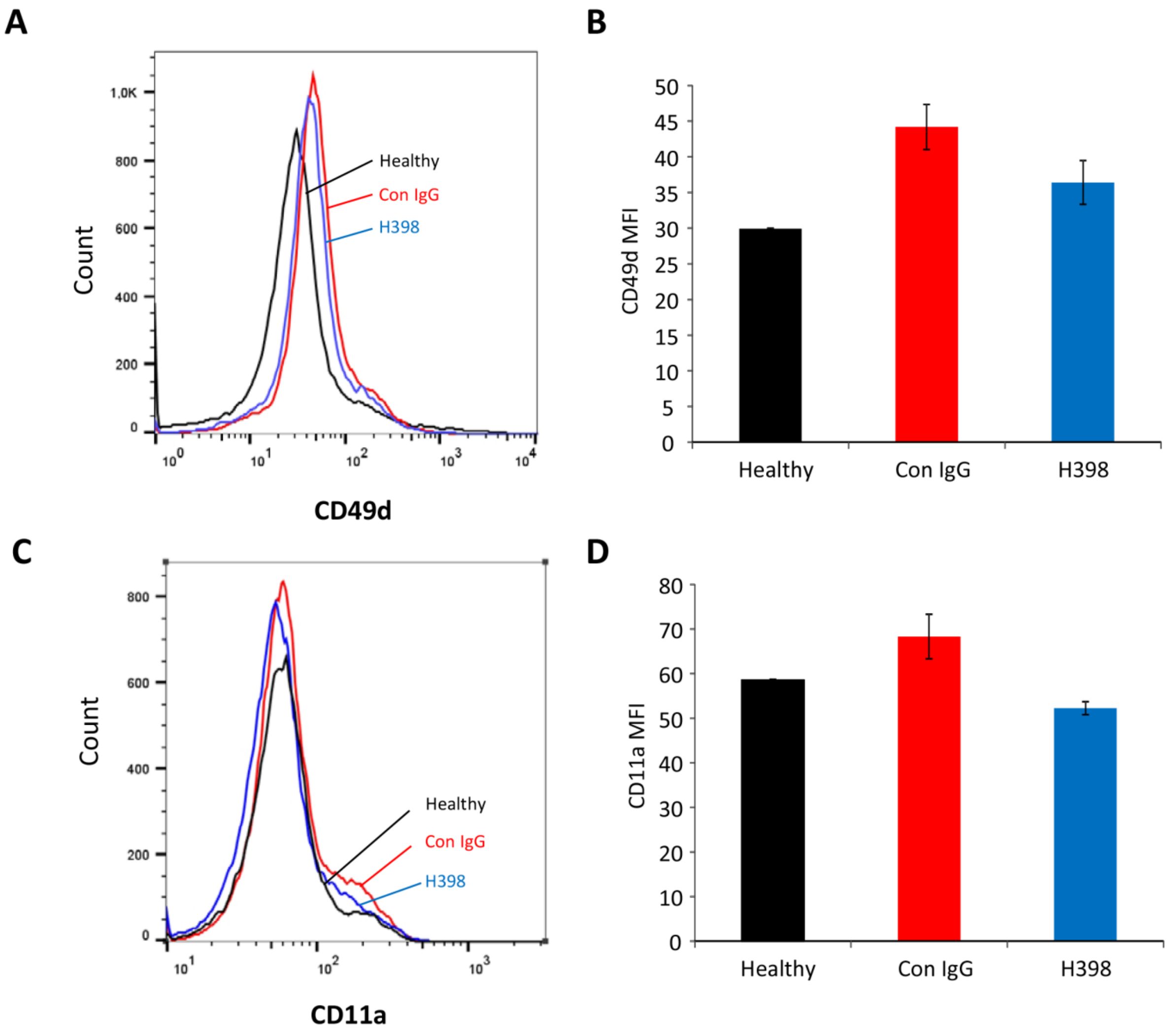

Supplementary Figure 2. Flow cytometry of T cells isolated from healthy hu/m TNFR1 ki mice, or mice at day 21 of EAE treated either with control IgG (Con IgG) or the TNFR1 antagonistic antibody H398. (A, C) Representative histograms of T cells labelled with antibodies against CD49d (the α chain of VLA-4, ligand for VCAM-1) or CD11a (αL integrin from LFA-1, ligand for ICAM-1). No significant differences were seen in the median fluorescence intensity (MFI) of either CD49d or CD11a. n = (healthy) 2, (Con IgG) 4, and (H398) 3.
